# Two Cases of White Globe Appearance in Autoimmune Atrophic Gastritis

**DOI:** 10.1155/2018/7091520

**Published:** 2018-10-30

**Authors:** Masaya Iwamuro, Takehiro Tanaka, Hiromitsu Kanzaki, Seiji Kawano, Yoshiro Kawahara, Hiroyuki Okada

**Affiliations:** ^1^Department of Gastroenterology and Hepatology, Okayama University Graduate School of Medicine, Dentistry, and Pharmaceutical Sciences, 2-5-1 Shikata-cho, Kita-ku, Okayama 700-8558, Japan; ^2^Department of Pathology, Okayama University Hospital, Okayama 700-8558, Japan; ^3^Department of Endoscopy, Okayama University Hospital, Okayama 700-8558, Japan

## Abstract

In this report, we described two patients with white globe appearance in autoimmune atrophic gastritis. Endoscopy revealed multiple white substances in the stomach in both cases. Biopsied specimens from the lesions contained dilated glands and showed a decrease in parietal cells. Intraglandular necrotic debris and carcinoma were absent. These results confirmed that white globe appearance can be observed in autoimmune atrophic gastritis. Moreover, microscopic features for white globe appearance observed in these cases were different from those reported previously in gastric cancer lesions and were similar to those observed for noncancerous stomach.

## 1. Introduction

White globe appearance has been reported to be characterized by a small (<1 mm) white lesion with a globular shape and microvessels overlying the white substance, reflecting the white substance existing underneath the gastric epithelium and subepithelial microvessels [1–3]. Detection of white globe appearance during esophagogastroduodenoscopy is clinically significant because this endoscopic feature is reportedly found within the margin of the cancerous gastric epithelium.

Although the white globe appearance is generally associated with the presence of gastric cancer, it can be rarely found in the noncancerous mucosa of the stomach. We have recently reported endoscopic pictures and detailed pathological features of white globe appearance in two patients without gastric cancer [4]. Herein, we reported white globe appearance detected in two cases of autoimmune atrophic gastritis, mainly focusing on the differences in pathological features between the two present cases and previously reported cases.

## 2. Case Report

### 2.1. Case 1

A 66-year-old Japanese woman was referred to the Department of Neurology for investigation of aphasia. The patient had no previous disease history and does not take any medications. Laboratory testing revealed decreased levels of vitamin B_12_ at 107 pg/mL (normal range: 257-989 pg/mL), for which she underwent esophagogastroduodenoscopy. Increased levels of rheumatoid factor at 38.9 IU/mL, hemoglobin A1c at 6.5%, and gastrin at 1,016 pg/mL were also noted. The number of red blood cells and the hemoglobin levels were within the normal ranges. She was positive for anti-intrinsic factor antibody and antiparietal cell antibody.

**Figure 1:**
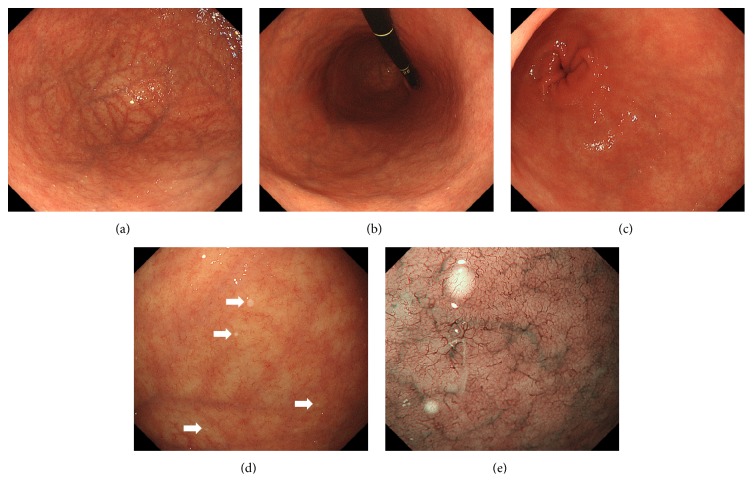
Esophagogastroduodenoscopy images of Case 1. Atrophy is predominantly observed in the gastric fornix (a) and body (b), while it is not evident in the antrum (c). Close-up observation of the gastric fornix shows multiple white substances (d). Magnifying observation with narrow-band imaging reveals microvasculature on its surface (e).

**Figure 2:**
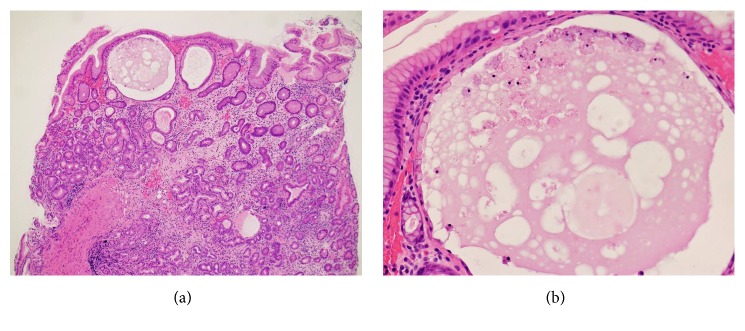
Pathology images of Case 1. Biopsy specimens taken from the gastric mucosa with white globe appearance show cystic dilatation of the gastric glands (hematoxylin and eosin staining, (a): ×4.2, (b): ×20).

Esophagogastroduodenoscopy revealed gastric atrophy predominantly in the fornix (Figure 1(a)) and in the body (Figure 1(b)), whereas atrophic changes were not evident in the antrum endoscopically (Figure 1(c)). Close-up observation of the gastric fornix showed multiple, slightly elevated, round, white substances (Figure 1(d)). Magnified observation with narrow-band imaging revealed microvasculature on its surface, suggesting deposition of the white substance within the mucosa (Figure 1(e)). Two biopsy samples were endoscopically taken from the fornix mucosa with the white substance. Three additional biopsies were done on the mucosa of the middle body, the lower body, and the antrum of the stomach, where the white substance was absent. Two biopsy specimens taken from the gastric mucosa that contained white substance revealed cystic dilatation of the gastric glands (Figure 2). In contrast to this, there was no cystic dilatation in the glands of the gastric mucosa specimens where the white globe appearance was not observed. A prominent decrease in parietal cells was also noted. Parietal cell protrusion was absent. There were no* Helicobacter pylori *pathologically. We diagnosed autoimmune atrophic gastritis based on the serology, endoscopy, and pathology results.

### 2.2. Case 2

**Figure 3:**
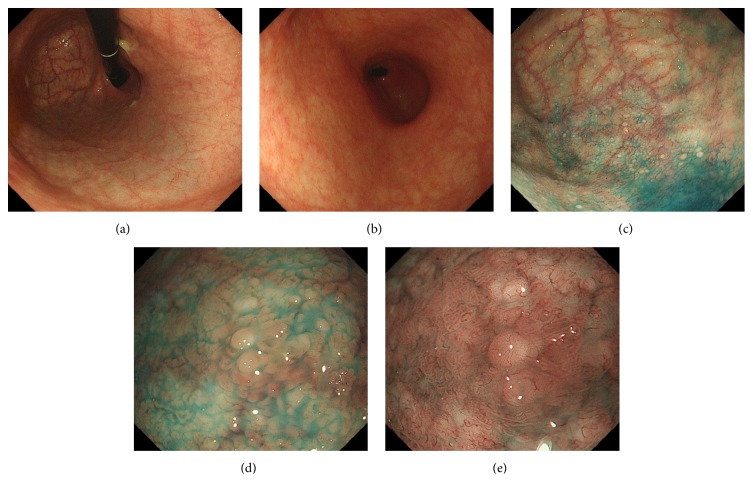
Esophagogastroduodenoscopy images of Case 2. Atrophy is more prominent in the fornix (a), compared to the antrum (b). Multiple white substances are seen in the gastric fornix and in the body after indigo carmine spraying (c, d). Microvasculature is also observed on magnified observation with narrow-band imaging (e).

**Figure 4:**
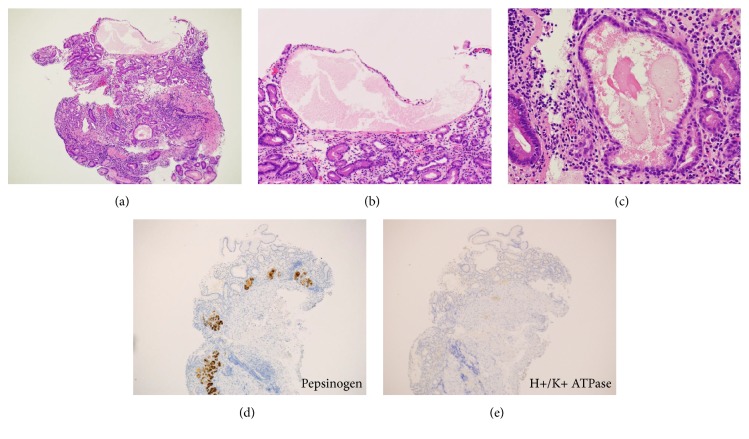
Pathology images of Case 2. Biopsy from the gastric mucosa shows cystically dilated glands (hematoxylin and eosin staining, (a): ×4.2, (b): ×10, (c): ×20). Immunohistochemical study shows that pepsinogen-positive cells ((d), ×4.2) are present, whereas H+/K+ ATPase-positive cells are absent ((e), ×4.2).

An 81-year-old Japanese woman underwent esophagogastroduodenoscopy for annual screening purposes. The patient had been taking amlodipine for hypertension. Although she had undergone appendectomy at 30 years of age, she had no other history of abdominal diseases. Esophagogastroduodenoscopy showed diffuse gastric atrophy; the atrophic changes were more prominent in the fornix (Figure 3(a)) than in the antrum (Figure 3(b)). After indigo carmine spraying, we noticed multiple, slightly elevated, round, white substances in the gastric fornix and body (Figures 3(c) and 3(d)). Microvasculature was also noted on its surface as seen on the magnified observation with narrow-band imaging (Figure 3(e)). Biopsy from the gastric mucosa showed cystically dilated gastric glands (Figures 4(a)–4(c)). Immunohistochemically, pepsinogen-positive cells were present (Figure 4(d)), whereas H+/K+ ATPase-positive cells were absent (Figure 4(e)).

Laboratory testing revealed normocytic anemia: hemoglobin was 10.2 g/dL, mean corpuscular volume was 92.5 fL, and mean corpuscular hemoglobin was 29.7 pg. Gastrin level was elevated at 392 pg/mL. Although the level of folic acid was decreased at 2.8 ng/mL, vitamin B_12_ level was within the normal range. The patient was positive for antiparietal cell antibody, while anti-intrinsic factor antibody was negative. She tested negative for urea breath test and* H. pylori* IgG antibody. Consequently, we diagnosed her as autoimmune atrophic gastritis.

**Figure 5:**
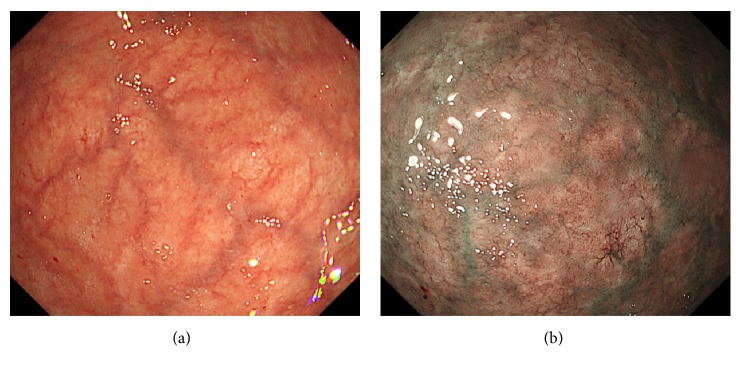
Esophagogastroduodenoscopy images of Case 2. Endoscopy performed 12 months later shows no white substances even in the gastric fornix ((a): white light, (b): narrow-band imaging).

The patient underwent esophagogastroduodenoscopy 12 months later. White substances were not detected in the gastric mucosa, even in the gastric fornix and body (Figure 5).

## 3. Discussion

Autoimmune atrophic gastritis, also known as type A gastritis, is an inflammatory disease affecting the gastric mucosa, wherein the parietal cells are destroyed by the patient's immune system. The progressive damage to the parietal cells decreases the ability to absorb iron and vitamin B_12_, which in turn causes pernicious anemia and neurological problems [5–7]. Since the diagnostic criteria have not yet been established, diagnosis of autoimmune atrophic gastritis is made based on laboratory testing, endoscopic examination, and biopsy results. Serologically, anti-intrinsic factor antibodies are highly specific for the disease, while the sensitivity is low. Conversely, antiparietal cell antibodies have high sensitivity but low specificity [8]. On esophagogastroduodenoscopy, atrophic changes are predominantly positive in the fornix and in the gastric body. On biopsy, chronic inflammation and oxyntic gland destruction are the most common histologic findings. We diagnosed the two patients with autoimmune atrophic gastritis, since they had these serological, endoscopic, and pathological features.

To our knowledge, this report is the first to describe the presence of white globe appearance in autoimmune atrophic gastritis. It was noteworthy that cystic dilatation of the gland was identified in the specimens of the gastric mucosa with white globe appearance in Case 1. Conversely, no cystic dilatation was present in the glands of the gastric mucosa specimens where the white globe appearance was not observed. This observation indicates that cystically dilated glands correspond to the endoscopically observed white globe appearance in the gastric mucosa. Another interesting observation from Case 2 is that white globe appearance was not identified on repeat endoscopy performed 12 months later. Therefore, this feature may spontaneously disappear during the course.

As previously described, white globe appearance in the stomach has been reported as an endoscopic feature indicative of cancer [1–3]. However, our earlier report showed detailed endoscopic and pathological pictures of white globe appearance observed in the noncancerous gastric mucosa and they revealed that the pathological features are different between cases with and without cancer lesions [4]. Pathologically, biopsy specimens taken from the white globe appearance with a cancer lesion contain eosinophilic materials with necrotic epithelial fragments within the lumen of the dilated glands; they are called intraglandular necrotic debris [1, 2, 9]. Meanwhile, specimens taken from the white globe appearance without cancer had cystic dilatation of the gastric fundal glands but no eosinophilic materials or necrotic epithelial fragments were identified [4]. For the present patients, we speculated that the deposited material was mucus in Case 2 and degenerated epithelial cells and mucus in Case 1. This report revealed that the pathological features of white globe appearance in autoimmune atrophic gastritis are somewhat similar to those observed for noncancerous stomach. However, a crucial difference between the white globe appearance observed in noncancerous stomach and that of autoimmune atrophic gastritis is that the former shows pseudoparietal cell hyperplasia with tongue-like protrusions of parietal cell cytoplasm (parietal cell protrusion) [4], while the latter shows a decrease or elimination of parietal cells via the autoimmune response.

Given that the white globe appearance in noncancerous gastric mucosa and that of autoimmune atrophic gastritis show similar pathologic features, several possible mechanisms can be hypothesized for the pathogenesis. First, obstruction of outflow from the glands within the gastric mucosa might induce such features. In autoimmune atrophic gastritis, inflammatory response to the parietal cells leads to the destruction of oxyntic glands. It is likely that such damage induces narrowing or collapse of the ducts, finally resulting in intramucosal cyst formation of the dilated glands. As reported previously, white globe appearance in noncancerous stomach shows parietal cell protrusion, which might cause outflow obstructions as well [4]. Thus, alteration of the duct structure may be the primary cause of the white globe appearance observed in noncancerous gastric mucosa and that of autoimmune atrophic gastritis.

The second hypothesis is that elevated levels of gastrin could induce white globe appearance. In autoimmune gastritis, hypochlorhydria from the loss of parietal cell mass leads to stimulation of the patient's antral G cells, which in turn results in gastrin secretion and hypergastrinemia [10]. As described above, the two patients showed elevated levels of serum gastrin. Meanwhile, patients with white globe appearance in noncancerous stomach have been taking acid secretion inhibitors [4]. Although we did not measure gastrin levels in these patients, such medications most likely induced hypergastrinemia. Therefore, elevated gastrin levels may play a part in inducing white globe appearance. However, concrete mechanisms between hypergastrinemia and formation of white globe appearance have not been elucidated yet.

In conclusion, we present two cases with white globe appearance observed in the stomach with autoimmune atrophic gastritis. The pathogenesis of white globe appearance has not yet been described; hence, further investigations are warranted.
